# Sound and Noise

**Published:** 1887-12

**Authors:** 


### AGGRAVATION FROM NOISE.

### SOUND AND NOISE.

As a subscriber has inquired after remedies having aggravation from noise, it is hoped the following notes may be of use. The list given is by no means complete:

Aconite.—Extremely sensitive to noise; music unbearable, goes through every limb; makes her sad. (Sabina, Theridion.)

[Note.—Arnica pains are worse from motion and noise; Theridion has, perhaps, the most characteristic aggravation from noise; study it.—Eds.]

AMM-C.—Painful sensitiveness of the dull ear to loud noise.

ANT-CRUD.—Sound of church bell is doleful.

ARNICA.—Great sensitiveness to loud sounds, spasms aggravated by noise, pains insufferable, drive him crazy; increased by every motion or noise.

ARSENIC.—Unusual sensitiveness to sound, talking of others aggravates the pains (also Mag-m. and Zinc).

ASARUM.—Scratching of linen or silk is insupportable.

BELLAD.—Extreme sensibility of hearing.

BORAX.—Very sensitive to slightest noise, as rumpling of paper, fall of door-latch, etc.

BRYONIA.—Intolerance of noise.

CALADIUM.—Sensitive to noise; the slightest noise startles from sleep.

COFFEA.—Hearing very acute, music has a shrill sound; aversion to noise, it hurts him.

CONIUM.—Painful sensitiveness of hearing, noise startles.

HYPERICUM.—Sensitiveness of hearing during menses.

IGNATIA.—Intolerance of noise, headache from noise, roaring in ears, relieved by music.

IPECAC.—Cannot stand least noise.

LACHESIS.—Crying and weeping from slightest noise.

LYCOPODIUM.—Every noise hurts her.

MURIAT-ACID.—Distant sounds cause headache; sound of voice unbearable.

NATR-CARB.—Music causes trembling.

OPIUM.—Acute hearing; clocks striking and cocks crowing at a great distance keep awake.

PHOSP-ACID.—Intolerance of noise, especially of music. (Aconite, Ambra, Cham., Nat-s., Nux-v., Sabina, Sepia.)

PTELEA.—Intolerance of loud talking.

SABINA.—So nervous, music is unbearable; it goes through bone and marrow. (Acon.)

SANGUINARIA.—Painful sensitiveness to sudden sounds, right ear worse, cannot bear to hear a person walking in the room, with extreme moroseness and nausea.

SECALE.—Undue sensitiveness of hearing, so that even the slightest sound re-echoed in her head and made her shudder.

SILICEA.—Painfully sensitive to noise.

SPIGELIA.—Hearing over sensitive in neuralgia and headache.

STRAMONIUM.—Very sensitive to noises, the least noise startles.

THERIDION.—Worse from least noise, every sound penetrates her whole body, especially the teeth. Nausea, vomiting, and vertigo from noise.

HEADACHE, worse from noise: Acon., Agar., Bapt., Bar., Bell., Bufo, Cact., Cic., Citr., Colch., Con., Ign., Iod., Mur-ac., Ptelea, Phosp., Phos-ac., Nit-ac., Spig., Zizia.

SPASMS, worse from noise: Ang., Arn., Ign.

*Mental states:*

SADNESS from music: Acon., Digit., Nat-s.

WEEPING from music: Creos., Graph., Nat-s., Thuja.

TALKING of people, can't bear: Amm-c., Ars., Con., Mag-m., Marum, Zinc.

FOOTSTEPS, noise of, is intolerable: Nit-ac., Nux-v.

